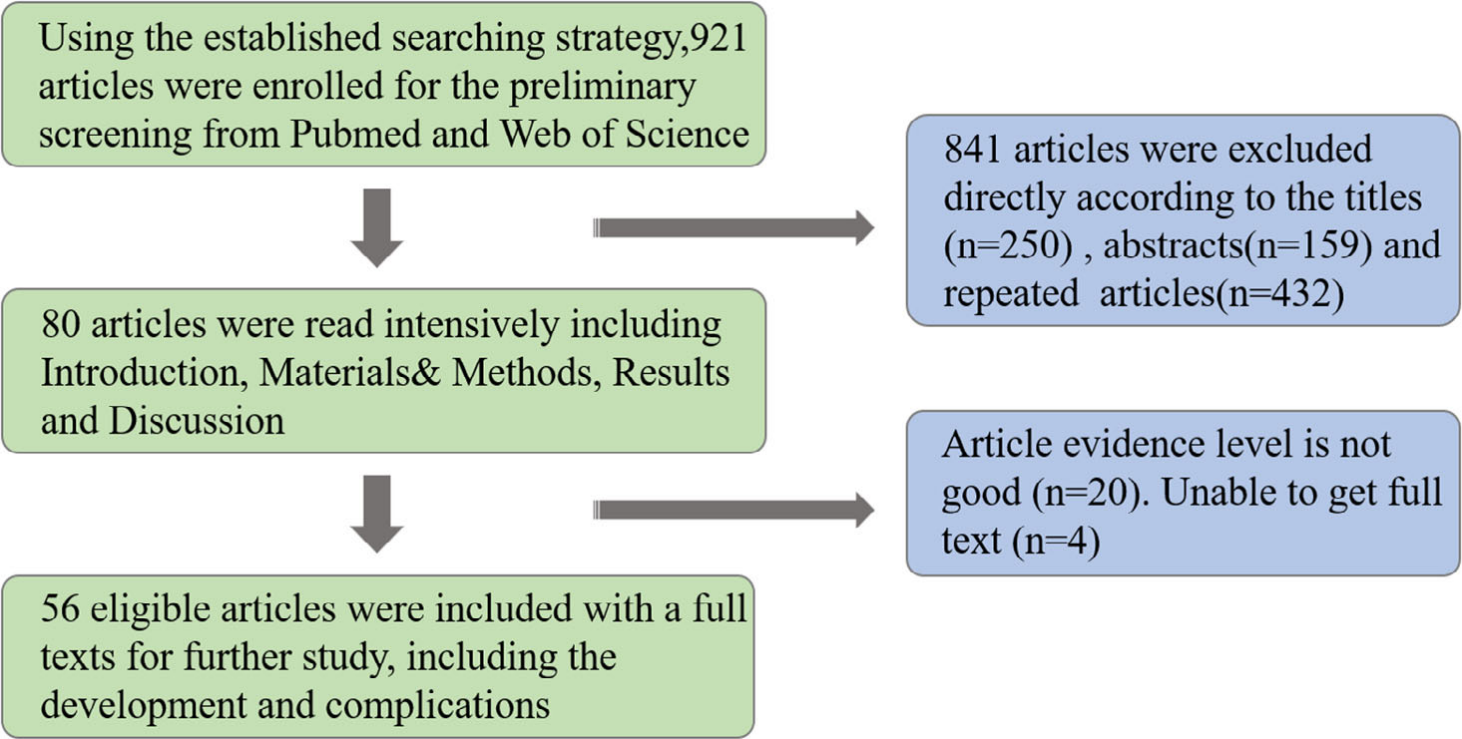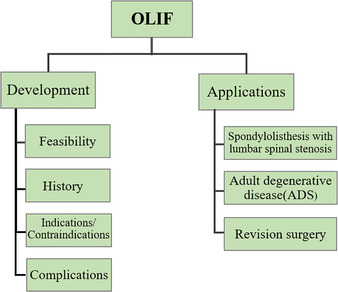# Development and Application of Oblique Lumbar Interbody Fusion

**DOI:** 10.1111/os.12625

**Published:** 2020-03-15

**Authors:** Renjie Li, Xuefeng Li, Hong Zhou, Weimin Jiang

**Affiliations:** ^1^ Department of Orthopaedics The First Affiliated Hospital of Soochow University Suzhou China

**Keywords:** Complications of OLIF, Development and application, Oblique Lumbar Interbody Fusion (OLIF)

## Abstract

The present study reviewed the relevant recent literature regarding the development and application of oblique lumbar interbody fusion (OLIF), with a particular focus on its application and associated complications. The study evaluated the rationality of this technique and demonstrated the direction of future research by collecting data on previous operative outcomes and complications. A literature search was performed in Pubmed and Web of Science, including the following keywords and abbreviations: anterior lumbar interbody fusion (ALIF), lateral lumbar interbody fusion (LLIF), direct lateral interbody fusion (DLIF), extreme lateral interbody fusion (XLIF), oblique lateral interbody fusion (OLIF), adjacent segment disease (ASD), and adult degenerative scoliosis (ADS). A search of literature published from January 2005 to January 2019 was conducted and all studies evaluating development and application of OLIF were included in the review. According to the literature, the indications for OLIF are various. OLIF has excellent orthopaedic effects in degenerative scoliosis patients and the incidence of bony fusion is higher than for other approaches. It also provides a better choice for revision surgery. It has various advantages in many aspects, but the complications cannot be ignored. As a new minimally invasive technique, the advantages of OLIF are obvious, but further evaluation is needed to compare its operation‐related data with that of traditional open surgery. In addition, more prospective studies are required to compare minimally invasive and open spinal surgery to confirm its specific efficacy, risk, advantages, learning curve, and ultimate clinical efficacy.

## Introduction

Spinal fusion was introduced by Hibbs and Albee in 1911, and has developed over the past more than 100 years to become one of the most commonly used surgical techniques in spinal surgery. Fusion techniques for lumbar diseases are diverse (e.g. anterior lumbar interbody fusion [ALIF], posterior lumbar interbody fusion [PLIF], transforaminal lumbar interbody fusion [TLIF], and extreme lateral interbody fusion [XLIF]), so as to meet the requirements of changes in indications, safety, and effectiveness. The PLIF technique requires an extensive dissection of the para‐spinal tissue as well as prolonged soft tissue retraction. Other disadvantages include significant blood loss and postoperative radiculopathy secondary to the prolonged retraction of the dural sac. XLIF needs to pass through the psoas major muscle, which can lead to injury of the lumbar plexus nerves.

Over the past two decades, the advent of minimally invasive approaches to the anterior lumbar spine has been one of the most significant developments in lumbar spine surgery. Minimally invasive surgery (MIS can ensure clinical efficacy and reduce trauma, intraoperative bleeding, bed rest time, and complications. In 2012, Silvestre reported a new minimally invasive technique called oblique lumbar interbody fusion (OLIF). The technique uses the anatomical space between the aorta/inferior vena cava (IVC) and the psoas muscle to access the disc space. The procedure is performed through the inter‐muscular space of the left inferior oblique, the internal oblique, and the transverse abdominal muscles into the extraperitoneal space. A working passage is placed between the abdominal aorta and psoas major muscles and the operation is performed through the passage. Unlike the traditional posterior approach, the lamina, the paravertebral muscles, and the facet joints are not destroyed in OLIF. Incision is made in the left abdomen and the intervertebral disc is reached through the gap between the abdominal aorta and the psoas major muscle. That is why OLIF has the advantages of less damage and bleeding, a lower rate of nerve injury, and faster recovery compared with traditional posterior surgery. As the OLIF technique continues to be developed, evidence‐based risk‐stratification systems are required to guide surgeons in choosing more optimal surgical approach. In addition, the scope of application and the curative effect needs to be further clarified.

The aim of this paper is to review the relevant recent literature regarding the development and application of OLIF, with a particular focus on the application and complications. The feasibility of this technique is evaluated and the direction of future research is explored by collecting data on previous operative outcomes and complications.

## Methods

A literature search was performed in Pubmed and Web of Science, including the following keywords and abbreviations: anterior lumbar interbody fusion (ALLF), lateral lumbar interbody fusion (LLIF), direct lateral interbody fusion (DLIF), extreme lateral interbody fusion (XLIF), oblique lateral interbody fusion (OLIF), adjacent segment disease (ASD), and adult degenerative scoliosis (ADS). A literature search from January 2005 to September 2019 was conducted and all studies evaluating development and application were included in the review. The inclusion criteria were: (i) periodical paper, academic paper, and review; (ii) article published recently in an authoritative magazine; and (iii) content of article is closely related to lumbar fusion or the application of related approaches, and is highly recognized by spine surgeons. The exclusion criteria were as follows: (i) low evidence level; (ii) unable to obtain full text; and (iii) repeat studies. If the article was in line with the topic mentioned above, the full text was accessed, and the article was read in detail, and included in this review.

## Development of Oblique Lumbar Interbody Fusion

### *History of Oblique Lumbar Interbody Fusion*

In 1932, Carpenter reported an anterior retroperitoneal fusion procedure, ALIF^1^. In 1997, Mayer reported an improved procedure for ALIF that involved using a retroperitoneal psoas major anterior approach in the L2–5 intervertebral space and an intraperitoneal approach in the L5–S1 intervertebral space. He referred to it as mini‐open ALIF. It prevents damage to the posterior ligament complex, traction of nerve roots, and dural tears, and decreases the incidence of adjacent joint degeneration^2^. Meanwhile, the effect is similar to that of traditional open surgery. However, an unsuitable operation may lead to complications such as reverse ejaculation and anterior vertebral vascular injury. The incidence of retrograde ejaculation was 7.4%^3^. In 2001, Pimenta^4^ first reported an approach of spinal fusion through the retroperitoneal space and the psoas major muscle using a tubular distractor. Ozgur *et al*. (2006)^5^ named it extreme lateral interbody fusion (XLIF). One year later, Knight *et al*.^6^ first reported the direct lateral interbody fusion (DLIF), which is similar to XLIF. OLIF was reported by Silvestre in 2012. Compared with XLIF and DLIF, the approach uses the anatomical space of the psoas major muscle and the muscle is not cut off. It can not only effectively avoid the risk of vascular injury caused by anterior surgery but also avoid injury of the lumbar plexus nerve caused by the damage to the psoas major muscle by DLIF. In addition, expensive neuromonitoring is not necessary during the operation and the incidences of hip flexion weakness and thigh numbness are lower than for XLIF and DLIF, which has attracted much attention by surgeons.

### *Anatomical and Imaging Study of the Feasibility of Oblique Lumbar Interbody Fusion Surgical Approach*

Davis *et al*. studied the anatomical structure of the L2–S1 lateral surgical pathway by using 20 cadaveric specimens, from 11 males and 9 females. They placed the specimen in a lateral position to measure the width of the surgical window ithen intervertebral space of L2–S1. The width was measured with the psoas in a static state and then with mild lateral retraction of the psoas. The study was divided into two parts. In the L2–5 oblique corridor, the distance was measured between the left lateral border of the aorta, or the nearest common iliac vessel if below the bifurcation, and the anterior ventral medial border of the psoas. In the L5–S1 oblique corridor, the distance was defined transversely from the midsagittal line of the inferior endplate of L5 to the medial border of the left common iliac vessel and vertically to the first vessel that crossed midline. In the static state, the average width of operation windows is 18.6 mm (L2–3), 19.25 mm (L3–4), and 15.0 mm (L4–5). When the psoas major muscle is stretched lightly, its average width is 25.5 mm (L2–3), 27.05 mm (L3–4), and 24.45 mm (L4–5). The L2–3 corridor increases by an average of 59.6%, and the L3–4 and L4–5 corridors by 43.96% and 58.97%, respectively. In addition, the horizontal and vertical widths of the L5–S1 operation windows are 14.75 mm and 23.85 mm, respectively. The gap between the abdominal aorta and psoas major muscle is 25.67 mm when the psoas major muscle is slightly stretched. The width of OLIF cage commonly used in clinic is 18 mm (CLYDESDALE Spinal, Medtronic, USA), which is accommodated by the space between the abdominal aorta and the psoas major muscle. However, for the limitation of rib arch and iliac crest, application in L1–2 and L5–S is undesirable. Davis also found that the left retroperitoneal space of the human body is greater than the right space. Molinares^7^ believed that preoperative images should be taken to determine whether the width is big enough and to find whether there are anatomical variations in the surgical space. Uribe^8^ divided the lumbar vertebrae into four zones between the anterior and posterior edges of the vertebral body in sagittal position. In lateral decubitus position, the lumbar plexus is distributed at the dorsal side of Zone IV and III (L1–2 and L3–4), and the intersection of Zones II and III (L4–5).

The cage is inserted into the posterior 1/3 position of the intervertebral space, increasing the height of the disc space and the foramen, which is the principle of indirect decompression of the spinal canal. The genitofemoral nerve runs along the psoas muscle, which should be treated with circumspection. Injury of the genitofemoral nerve may result in pain in areas like the inguinal region, the scrotum, the labia vulvae, and the dyskinesia of the testis muscle. It is commonly believed that the abdominal aorta and the inferior vena cava (IVC) are in front of the vertebral body. However, for OLIF, a delicate minimally invasive procedure, the term “front of vertebral body” is clearly not specific enough. The positions of the abdominal aorta and the IVC vary in different individuals and segments. In fact, it can be seen at the cross‐section of the MRI that these large vessels are not entirely in front of the anterior tangent of the vertebral body. It has been reported^9^ that the distribution of the abdominal aorta at L1–5 may cover part of Zone I, while on the right side of the vertebral body, the inferior vena cava trunk may also partly cover Zone I. Therefore, any sharp manipulation and cage placement in Zone I is dangerous.

**Figure 1:**
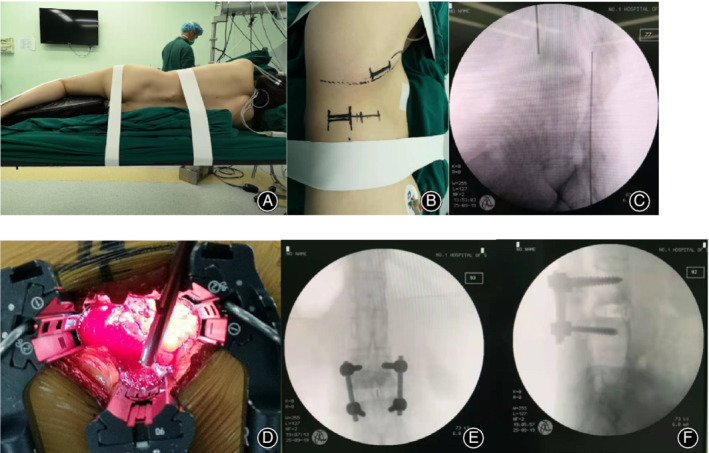
A 37‐year‐old female patient has had ankylosing spondylitis for 18 years. (A)–(C) Preoperative position placement and target level confirmation. (D) The exposure of the operative field. The images taken after the operation are shown in (E) and (F).

In addition, the approach may be affected by the shape of the psoas muscle. On the concave side of scoliosis patients, the space between the vessels and the psoas muscle decreased, which is not conducive to the establishment of the OLIF surgical pathway. Hence, attention should be paid to the shape before the operation. It is difficult to perform the operation if the muscle is rising (rising psoas sign). Besides, the space between the psoas muscle and the psoas quadratus muscle increases in some patients, which could lead to mistaking the gap for the gap between the artery and the psoas muscle. Different positions have an influence on the shape of the psoas muscle. In the right decubitus position, the left psoas major muscle is affected by gravity and is close to the vertebral body. Hip flexion and knee flexion will also relax the psoas major muscle, increasing the cross‐sectional area of the psoas major muscle. (Fig. 1).

### *Indications and Contraindications of Oblique Lumbar Interbody Fusion*

**Figure 2:**
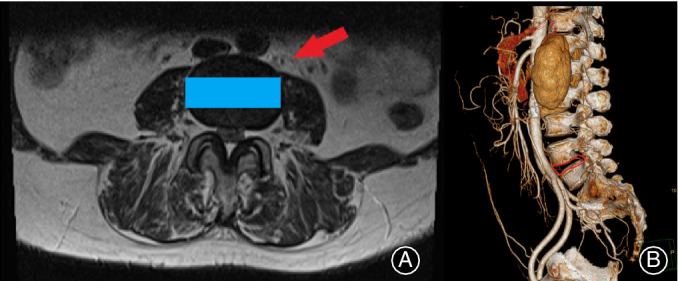
(A) Arrow indicates the route to the intervertebral disc and the rectangle shows the position where the cage is inserted. Cross‐section of the MRI determines if it is suitable to perform oblique lumbar interbody fusion (OLIF). (B) CTA is used to find whether there is abnormal vascular malformation to decrease the incidence of vascular injury.

**Figure 3:**
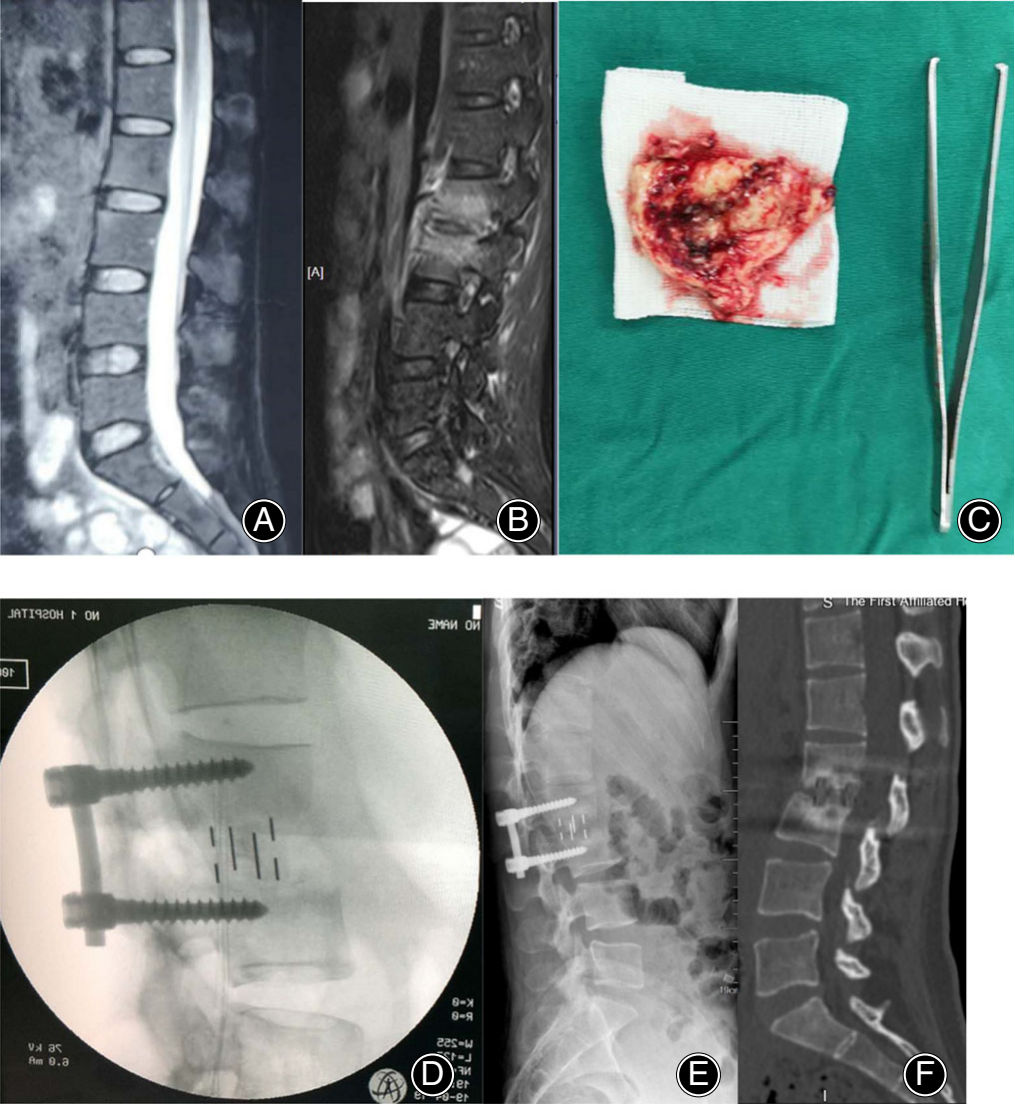
The preoperative sagittal T2‐weighted MRI (A, B) of a 31‐year‐old female patient showed the disruption of intervertebral space 1 week and 2 months after the onset of low back pain, respectively. (C) The disrupted intervertebral disc. The lateral (D–F) radiographs and CT showed oblique lumbar interbody fusion at L2–3 levels intraoperatively, 3 and 6 month postoperatively. Bony fusion was achieved.

**Figure 4:**
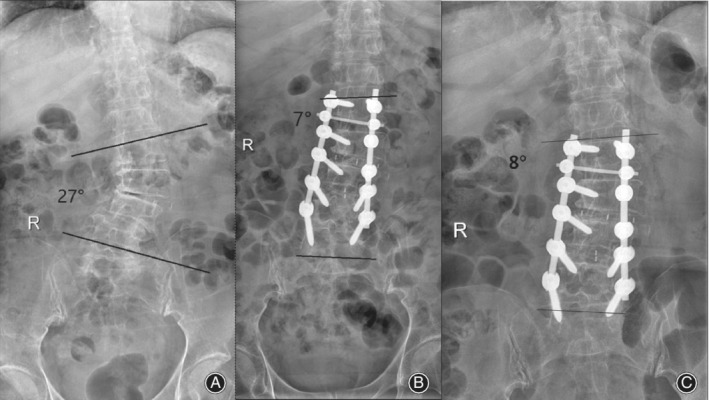
A 68‐year‐old woman underwent oblique lumbar interbody fusion (OLIF). The anteroposterior (A) X‐ray radiographs show scoliosis deformity and the Cobb angle is 27°. The X‐ray radiographs (B) and (C) at 1 week and 5 months postoperatively, respectively, show that the Cobb angle decreased 19° and the deformity was rectified remarkably.

Indications for OLIF are limited, including discogenic low back pain, lumbar degenerative scoliosis, type I‐II lumbar spondylolisthesis, lumbar instability, lumbar tuberculosis, lumbar revision, and mild‐to‐moderate spinal stenosis (Figs 2, 3, 4). The endplate can easily be damaged during preparation for the operation, causing iatrogenic subsidence, which is associated with the bone quality of the patient. Therefore, for patients with osteoporosis, the literature suggests that bilateral transpedicular fixation with screws can assist in avoiding the implant subsidence for patients whose bone mineral density T value is less than −1.0.

The posterior ligament complex is retracted by increasing the intervertebral height. Therefore, in patients with osseous spinal canal stenosis, congenital spinal canal stenosis, and intraspinal space‐occupying lesions, such as prolapsed nucleus pulposus, tightening the ligament is useless. Besides, the protrusion is out of the ligament and cannot be retracted by tightening the ligament. In addition, the symptoms will not be relieved by indirect decompression in patients with severe spinal stenosis. Meanwhile, OLIF is not recommended for patients with spontaneous fusion of intervertebral space or posterior facet joints. According to the minimally invasive spinal deformity surgery (MISDEF) classification proposed by Mummaneni^10^, type III adult deformity requires osteotomy and three‐column and thoracic spine fusion, which is not suitable for OLIF.

### *Complications of Oblique Lumbar Interbody Fusion*

According to the anatomy and surgical procedures, we divide the complications into two parts, including intraoperative and postoperative complications. The former include abdominal vascular injury, endplate damage, the cage being embedded, and vertebral fracture. Postoperative complications include cage sedimentation or shifting, transient psoas weakness, pain and numbness in front of the left thigh, lesion of the sympathetic chain, lilac crest pain, transient quadriceps weakness, left lower abdominal pain, incomplete ileus, and contralateral nerve root injury.

#### *Abdominal Vascular*

**Figure 5:**
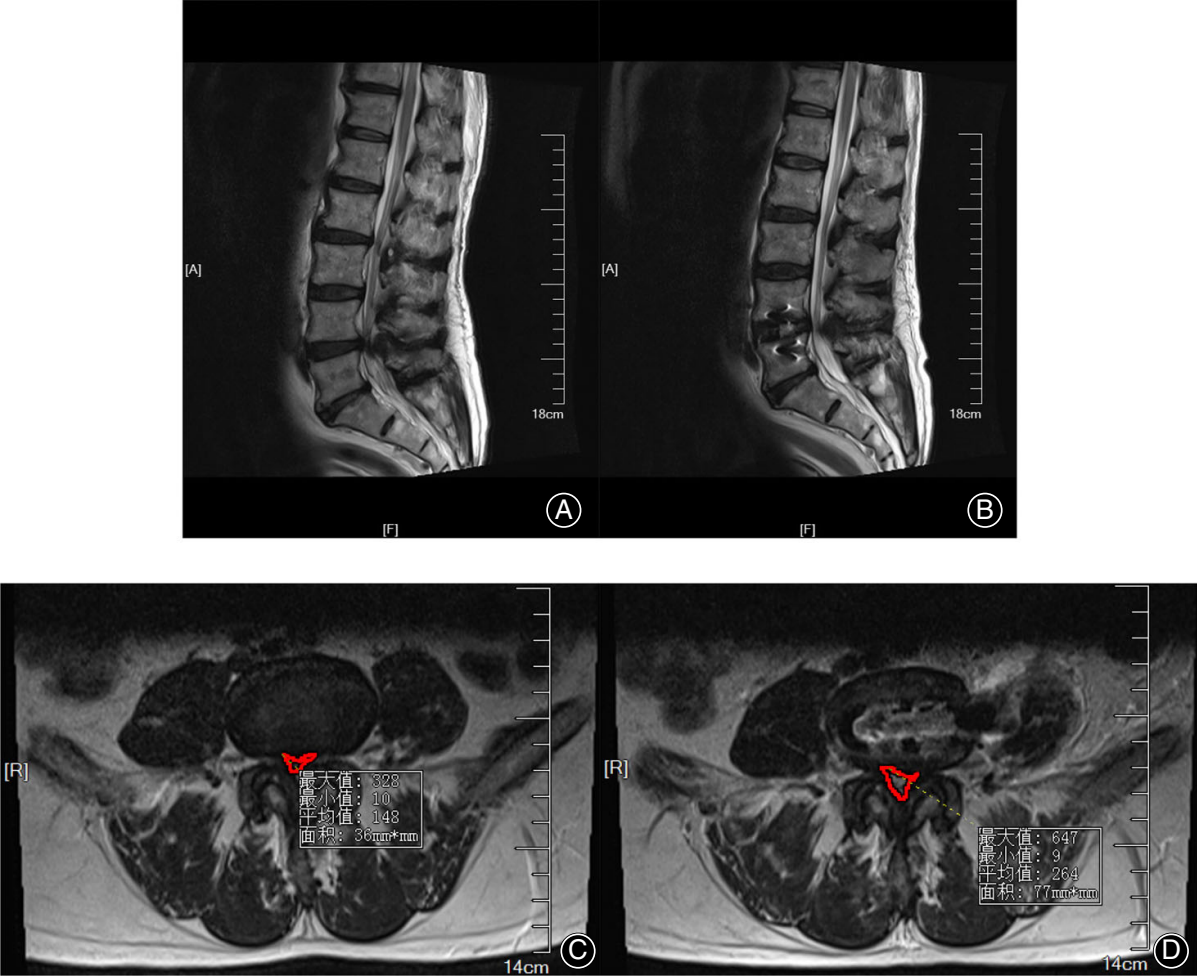
Pictures (A) and (C) were taken preoperatively and pictures (B) and (D) were taken after the surgery. The comparison of images showed that a significant increase in the spinal canal area after the operation, showing good effect of indirect decompression of the spinal canal.

Abdominal macrovascular injury is the most serious intraoperative complication of OLIF. Arterial injury rates in OLIF were previously reported as 0.3%–2.4%^11^. Once it occurs, it has serious consequences. The local anatomical relationship of tissues and organs should be clearly understood. The abdominal aorta is located on the left anterolateral side of the lumbar spine and the vena cava is located on the right anterolateral side. CTA of abdominal vessels is routinely performed before surgery to find whether there is anatomical variation of vessels in the operation area (Fig. 5). Molinares^7^ suggested that a width of gap less than 1 cm between the psoas muscle and the anterior vertebral artery is not suitable for this approach. In addition, when breaking through the contralateral annulus fibrosus, the breakthrough point should not be too close to the front of the vertebral body where the inferior vena cava is located, which is easy to rupture and difficult to repair.

### *Urethral Injury*

Urethral injury is an intraoperative complication with lower incidence than in other surgeries, including pelvic surgeries for colon and gynecologic surgery. Various authors coincide in noting that the level of greater risk for a ureter lesion is L2–L3^46^. The ureter can be easily injured during any stage of the retroperitoneal corridor dissection and the placement of the tubular retractor^15^. The ureter is located behind the peritoneum and descends vertically into the pelvis through the medial front of the psoas muscle. According to Javier^15^, it is localized anterior to the psoas muscle in 90.4% of cases, and lateral to the vertebral body in 16% of cases. This hidden symptom is difficult to diagnose. The possibility of a ureter lesion should be considered in cases of abdominal pain, fever, leukocytosis, or abdominal distention. Kubota^12^, ^13^ demonstrated that delayed contrast‐enhanced CT and retrograde urography are useful in diagnosing the injury. Urethral injury may be avoided by the complete retraction of the retroperitoneal fatty tissue before starting the discectomy and the anterior mobilization of the ureter. Besides, patients with urological diseases or retroperitoneal tumors are not suitable for OLIF. The anatomical structure should be understood well. The extraperitoneal fat is pushed to the ventral side. The operation must not be conducted through fat, which would risk injury to the ureter. Preoperative catheterization and intraoperative observation of urine color are performed routinely. If urethral injury potentially exists, it is necessary to remove the dilation passage and carefully observe whether there is a continuous flow of liquid in the retroperitoneal. If the diagnosis is clear, the ureter should be repaired instantly (Table 1).

**Table 1:** Complications reported in the literature

Author	Size	Incidence (intra/postoperative complications; %)	Complications reported (*N* = patients)	Follow‐up (months)
Fujibayashi^42^, 2017	28	Intra = 0 Post = 28.6% (8/28)	Transient weakness of hip flexion = 2 Transient thigh pain/numbness = 6	‐
Sato^24^, 2017	20	Intra = 5% (1/20) Post = 15% (3/20)	Segmental artery injury = 1 Thigh pain/numbness = 1 Cage subsidence = 2	12
Ohtori^36^, 2015	35	Intra = 2.9% (1/35) Post = 17.1% (6/35)	Transient thigh pain/numbness = 3 Cage subsidence = 1 Quadriceps weakness = 1 Thigh pain = 1 Segmental artery injury = 1	14.5
Mehren^14^, 2016	812	Intra = 0.4% (3/812) Post = 3.3% (27/812)	Infection = 5 Hematoma = 11 Paralytic ileus = 2 Vascular Injury Vena iliaca communis left = 1 Vena iliaca communis right = 1 Aorta = 1 Nerve irritation = 3 Ilioinguinal and genitofemoral nerve = 1 Lumbar plexus = 2 Iliac crest pain = 6 Sensory deficits = 1	‐
Molloy^43^, 2016	64	Intra = 4.7% (3/64) Post = 31% (20/64)	Transient motor evoked potentials deficits = 3 Revision Procedures = 3 Wound Complications = 2 CSF leaks = 4 Ileus = 8 Pulmonary emboli = 3 Temporary failed catheter removal = 3	21.6
Grangnaniello^44^, 2016	21	Intra = 0 Post = 38% (8/21)	Weakness of hip flexion = 2 Extensor hallucis longus weakness = 1 Lateral cutaneous nerve palsy = 2 Sympathetic chain symptoms = 1 Psoas abscess = 1 New sacro‐iliac joint = 1	8.57
Jin^18^, 2018	21	Intra = 0 Post = 33.3% (7/21)	Leg paresthesia = 2 Local hematoma = 1 Abdominal ileus = 4	‐
Chang^21^, 2017	1	Intra = 0 Post = 1	Ventral dural injury = 1	24
Lee^13^, 2017	1	Intra = 0 Post = 1	Ureter injury	2
Abe^41^, 2017	155	Intra = 26.5%(41/155) Post = 22%(34/155)	Endplate injury = 29 Segmental artery damage = 4 Peritoneal laceration = 3 Other vessels injury = 2 Pleural laceration = 2 Ureteral injury = 1 Psoas weakness = 21 Surgical site infection = 3 Reoperation = 3 Breakage of the lumbar interbody fusion cage = 2 Surgical instrument failure = 2 Spinal nerve injury = 1 Cauda equina injury = 1 Postoperative death = 1	‐
Woods^45^, 2017	137	Intra = 4.4% (6/137) Post = 8.8% (12/137)	Cage subsidence = 6 Ileus = 4 Vascular injury = 4 Blood transfusion = 2 Superior mesenteric artery syndrome = 1 Retrograde ejaculation = 1	6

#### *Lesion to Sympathetic Chain*

In the current reports, the incidences of sympathetic chain injury were varied, ranging from 1.7% to 8.7%^44^, ^47^, ^49^, ^50^, ^51^. Between the anterior longitudinal ligament as a medial landmark and the psoas muscle as a lateral landmark is the natural “safety” corridor, which is covered by the fibers of the sympathetic chain^14^. Despite the lesion being reported frequently in the literature, little technical advice is a to avoid its injury^18^, ^47^, ^48^. Digital infrared thermal imaging and physical exploration can be used to clearly identify a sympathetic chain injury. Javier suggests a tubular stretcher being placed behind the sympathetic chain to decrease the incidence of traction lesions. Gragnaniello^44^ demonstrated that the sympathetic trunk has to be mobilized by smooth retractor blades; even sacrifice produces only warming of the affected leg that is unnoticed by patients.

#### *Cage Sedimentation*

Several factors can account for cage sedimentation, which is related to the technique, the implant material, and the bone quality of the patient^52^, ^53^, ^54^. The incidence of sedimentation ranges from 2.9% to 10%^20^, ^36^, ^45^. It is important to bear in mind that the endplate is concave and resistant peripherally and weaker centrally, which means that only the edge of the cage can support the endplates immediately after implantation. When patients stand up, the cage will be subjected to the stress of the endplate, which leads to a certain degree of sedimentation, so that the endplates have better contact with the cage, resulting in the loss of fusion segment disc height. Cage sedimentation can be classified into two types: those that are visible in postoperative imaging, and those that have specific clinical symptoms which can be explained by the loss of indirect decompression efficacy, including axis pain and recurrence of neurological symptoms. Avoidance of an aggressive endplate preparation is recommended^55^. The endplate damage without posterior pedicle screw fixation accounts for the sedimentation^14^. Therefore, posterior fixation needs to be applied in patients with endplate damage. Improper choice of cage model, obese patients, vigorous postoperative activity, and internal fixation methods (including lateral unilateral fixation, posterior fixation, and stand‐alone fixation), play important roles in sedimentation. Liu *et al*. (2007)^16^ observed 67 patients who underwent OLIF. Eighteen cases with posterior fixation and no cage sedimentation arose occurred during follow up. In the cases of unilateral fixation and stand‐alone fixation, the incidence of cage sedimentation was 3.85% and 26.09%, respectively. Obviously, posterior fixation decreases the incidence of sedimentation but the increased cost, prolonged operation time, and greater damage to the body should be taken into consideration. All of the disadvantages are avoided in patients with stand‐alone fixation. Factors in maintaining the stability of the cage include: bone quality and integrity of endplate, proper size, elasticity force of the anterior and posterior longitudinal ligaments, and tensile stress of the posterior ligament complex. Thus, the patients who are not consistent with indications of stand‐alone fixation are as follows: those with endplate damage, osteoporosis, lumbar instability, isthmic spondylolisthesis, lumbar spondylolisthesis of degree II and above, and multi‐segment fusion. OLIF combined with lateral single screw fixation was used in patients with single segment degenerative disease, degree I spondylolisthesis, so as to achieve the aims of less damage to the body and better efficacy.

Li *et al*.^17^ reported an intraoperative complication rate of 1.5% and a postoperative complication rate of 9.9% among a total of 1453 patients undergoing OLIF. Vascular injury is one of the most common intraoperative complications. Jin *et al*.^18^ compared patients with MIS‐DLIF and MIS‐OLIF, including 22 patients with MIS‐DLIF and 21 patients with MIS‐OLIF, all of whom had single segment degeneration. In patients with MIS‐DLIF, 13.6% had long‐term complications (i.e. which lasted more than 30 days). In contrast, only 2 cases of anterior thigh numbness and 1 case of local hematoma were reported in MIS‐OLIF patients. Those patients recovered 5–7 days after surgery.

In addition, there was no significant difference in operation time, intraoperative bleeding volume, and hospitalization time between the two groups, and the clinical effect was similar. Abe^41^ reported that in 155 patients, the incidence of complications was 48.3%; 44.5% of these were intraoperative complications. The most common complications were endplate fractures. The others included short‐term psoas major weakness, short‐term neurological symptoms, segmental artery injury and incision infection, as well as 1 case of each of the following: ureteral injury, nerve root injury, and horsetail injury. Only 4.7% of patients experienced postoperative complications. Kaiser^19^ counted 51 OLIF patients, of which 3.9% had intraoperative complications, including vascular and dural tears; 17.6% of patients had postoperative complications, including transient intestinal obstruction, retroperitoneal hematoma, urinary tract infection, wound infection, and nerve root pain. Kim^20^ retrospectively analyzed 29 patients undergoing OLIF. A total of 8 cases of sedimentation were reported in 37 segments and 4 cases of lumbar plexus traction pain were relieved within 4 weeks after the operation. There were 4 cases of lumbar sympathetic nerve chain injury. Change^21^ reported a case of ventral dura mater injury during endplate preparation. Lee^13^ reported an intraoperative urethral injury.

### *Clinical Application of Oblique Lumbar Interbody Fusion Technology*

#### *Oblique Lumbar Interbody Fusion for Adult Lumbar Spondylolisthesis with Lumbar Spinal Stenosis*

Lumbar spondylolisthesis is a common clinical disease, with an incidence of 5.9%. The ratio of males to females is approximately 1:221. The incidence is increasing year by year, which is accompanied by secondary spinal stenosis. The clinical manifestations are chronic low back pain and intermittent claudication. The symptoms of most patients after conservative treatment are not significantly alleviated. Surgical treatment is needed to relieve pain and achieve maximum functional improvement. Transforaminal lumbar interbody fusion (TLIF) is the most commonly used surgical method, which can not only effectively decompress and reduce lumbar spondylolisthesis but can also be fixed with pedicle screws. However, the injuries to paravertebral muscles can be serious and chronic low back pain may occur after the operation^22^. OLIF can fully open the intervertebral space for indirect decompression due to the large fusion cage. The height of the intervertebral foramen, the posterior height of the intervertebral space, and the area of the spinal canal increase significantly after the operation. In addition, sagittal imbalance can lead to forward movement of the center of gravity, resulting in excessive fatigue of related muscles, and increasing the stress of the lumbar spine. OLIF with an angular fusion cage can effectively restore lumbar lordosis and maintain sagittal balance of the lumbar spine. It can also increase the contact area of the bone graft. Its immediate stable support also provides a good environment for bone fusion, resulting in a high rate of bone fusion.

Fang *et al*.^23^ followed up 20 patients with lumbar spondylolisthesis treated with OLIF for half a year, and found that the degree of lumbar spondylolisthesis was well recovered. The retrolisthesis index (RI) was 23.5% ± 7.4%, and decreased to 4.2% ± 3.15% at the last follow‐up. The height of the intervertebral space increased from 6 ± 3.6 mm preoperatively to 10.8 ± 1.7 mm. The lumbar lordosis increased from 39.2° ± 8.4°to 45.0° ± 7.8°. CT showed that the size of the intervertebral foramen increased from 140.6 ± 36 mm^2^ to 179.8 ± 35.6 mm^2^ after the operation. MRI showed that the size of intervertebral foramen and the area of the dural sac increased from 78.1 ± 31.2 mm^2^ and 73.4 ± 29.3 mm^2^ before the operation to 141.7 ± 29.5 mm^2^ and 124.5 ± 26.6 mm^2^ after the operation, respectively. The visual analog scale (VAS) score of low back pain decreased from 6.7 ± 2.6 to 1.4 ± 1.1. The VAS score of lower limb pain decreased from 6.3 ± 2.7 to 1.0 ± 1.2. The data showed that OLIF is effective in the early stage of lumbar spondylolisthesis with secondary spinal stenosis, with minimal trauma and accurate reduction of vertebral slippage. The height of the intervertebral space can be restored effectively. Sato^24^ followed up 20 patients with lumbar degenerative spondylolisthesis. Preoperative and postoperative imaging data showed that the diameter of the MRI axis spinal canal increased from 12.4 mm to 13.9 mm, and the area of spinal canal increased from 93 mm^2^ to 113 mm^2^. The diameter of the sagittal MRI spinal canal increased from 8.9 mm to 11.0 mm. The intervertebral height increased from 6.3 mm to 10.2 mm. In addition, the literature reported a total of three kinds of complications. There were 2 cases of cage sedimentation, 1 case of thigh pain and numbness, and 1 case of segmental artery injury. Thigh numbness and pain diminished within 2 weeks of surgery.

Posterior lumbar interbody fusion is a classic operation for lumbar spondylolisthesis. Liu *et al*. (2017)^25^ compared PLIF with OLIF based on the concentration of CRP and CK in serum. Under the same anesthesia and medication after the operation, the OLIF group had less influence on the internal environment of the body after the operation than the PLIF group. The perioperative indicators of the average incision length, intraoperative bleeding volume, and postoperative hospital stay of the two groups have been studied in the literature, and the advantages of OLIF have been found to be great. It is clear that the reason for the conclusion is that the PLIF requires extensive dissection of the multifidus muscle, laminectomy, and facet process, resulting in spinal instability and scar adhesion. However, OLIF has defects which need to be improved^26^. First, repeated fluoroscopy is needed for the treatment of intervertebral discs. The patients receive X‐ray radiation longer than is the case for PLIF and TLIF. Second, a larger cage may damage the superior nerve roots on the oblique side of the Kambin triangle. In addition, the instruments for the treatment of intervertebral disc tissue still need to be improved, which can easily cause incomplete removal of the endplate structure, leading to non‐fusion of bone grafts and even the possibility of cage collapse.

### *Oblique Lumbar Interbody Fusion for Adult Degenerative Scoliosis*

Adult degenerative scoliosis (ADS) refers to new scoliosis deformity after the maturation of the skeleton, which has the characteristics of older age, longer course of disease, and more complications. The Cobb angle is more than 10°. The pathogenesis is multifaceted, including disc degeneration, vertebral compression fracture, osteoporosis, osteoarthritis, and other factors leading to the disorder of the sagittal plane of the spinal corona. The most common symptoms include low back pain, intermittent claudication, radicular pain, and other clinical symptoms, which seriously affect the quality of life. In some patients, surgical treatment is superior to conservative treatment. The purpose of surgery is reconstruction of spinal and decompress the spinal canal. At present, XLIF and DLIF are widely accepted for the treatment of complex ADS, including open intervertebral space, indirect decompression, and bone graft fusion, as well as secondary posterior internal fixation. However, the operation may damage the psoas major muscle and the lumbar plexus nerve deeply buried in the psoas major muscle. The risk of nerve injury after the operation is high, especially in the L4–5 gap. The incidence of sensory abnormalities and thigh pain after surgery is still as high as 25%–75% and 23%–60%, respectively^27^, ^28^, ^29^, even if neuromonitoring is used. Nohara^30^ have shown that the anterior approach is more effective than the posterior approach in correcting deformities. The Cobb angle of ADS is usually small^31^, which can be corrected effectively by inserting the cage into the intervertebral space in parallel. Degeneration of the intervertebral disc and narrowing of the intervertebral space in ADS result in shrinkage of the articular capsule and the ligamentum flavum, dislocation of articular process joints, and secondary stenosis of the central canal, the lateral recess and the intervertebral foramen. The anterior and posterior fibrous rings and longitudinal ligaments can be tightened after a large‐size cage is inserted, and the cage can be firmly fixed. Sharma *et al*.^29^ followed up 43 patients with scoliosis who underwent OLIF and reported that the coronal Cobb angle was corrected to 3.75° on average. Anand^32^ and others suggested that the coronal Cobb angle could be corrected from 22° to 7° after an operation. A 68‐year‐old woman underwent OLIF and the deformity were rectified remarkably. (Fig. 3).

Glassman *et al*.^33^ showed that the sagittal force line of ADS patients was closely related to clinical symptoms and quality of life (Oswestry disability index [ODI] score, SF12). According to the scoliosis research society–Schwab classification of adult scoliosis proposed by Schwab^34^, the three revised parameters of the sagittal vertical axis (SVA, cm), pelvic tilt (PT, °), and pelvic incidence‐lumbar lordosis (PI‐LL, °) are considered to be closely related to the quality of life. Some scholars^35^ believe that SVA <50 mm, PT <20°, and LL‐PI <10°are attributed to the thresholds of health. They can be used as important references for the surgeon to evaluate the sagittal balance of ADS patients before surgery and to formulate the operation plan. PI‐LL represents the severity of local malformations. SVA represents the overall spinal force line, and PT represents the compensatory mechanism of individuals. Ohtori *et al*.^36^ performed OLIF on 12 patients with scoliosis. The imaging data revealed that SVA decreased from 140 mm to 27 mm, PT decreased from 37 to 23°, PI‐LL decreased from 41° to 8°, the coronal Cobb angle decreased from 42° to 5°, and LL increased from 6 to 37°. Postoperative pain scores improved. Kim *et al*.^35^ performed OLIF on 32 patients with scoliosis deformity. SVA decreased from 136.6 mm to 29.4 mm, LL increased from 5.79° to 46.54°, the Cobb angle improved from 21.5° to 9.6°, and the fusion rate reached 84%. As a new technology, OLIF has excellent orthopaedic effect in scoliosis patients.

### *Oblique Lumbar Interbody Fusion for Revision Surgery*

Adjacent segment disease (ASD) is a common complication following spinal fusion, which leads to a series of symptoms such as low back pain and nerve root compression^37^, ^38^. According to the literature, risk factors for ASD include age >60 years, menopausal women, osteoporosis patients, and previous lumbar interbody fusion patients. Surgical treatment is needed when the pain is severe and conservative treatment is not effective. Surgical procedures include decompression alone, decompression and fusion, and artificial disc replacement. OLIF treatment of ASD patients is rarely reported in the literature. Phan^39^ reported a case of OLIF revision in 2015. The patients with L2–3 incompatibility after L2–4 TLIF were treated with the oblique lateral approach for intervertebral fusion. The pain was relieved immediately and no complications occurred. It is preliminarily confirmed that the revision operation can be used as an indication for OLIF. Zhu *et al*.^40^ followed up 17 patients with OLIF and 19 patients with PLIF. In the OLIF group, the operation time, the intraoperative bleeding volume, the time bedridden, and the hospitalization time were significantly shorter than in the PLIF group. The symptoms of lower limb pain in the OLIF group were milder than those in the PLIF group within 1 week after the operation. Considering the retroperitoneal approach, there was no need to open the vertebral canal and pull the nerve root. No significant difference was found in long‐term VAS and ODI scores. In addition, patients with ASD often have a history of lumbar spine surgery, which is difficult to perform again because of severe tissue adhesion during revision. OLIF can avoid adhesions by using a retroperitoneal approach, shorten the operation time, decrease trauma to the body, and improve the patient's tolerance to surgery.

### *Summary and Prospects*

Minimally invasive spinal surgery has made tremendous progress, and is a rapidly developing field. As a new minimally invasive technique, its advantages are obvious, but further evaluation is required to compare its operation‐related data with that of traditional open surgery. In addition, more prospective studies are needed to compare minimally invasive and open spinal surgery to confirm the specific efficacy, risks, advantages, learning curve, and ultimate clinical efficacy. Minimally invasive surgery has become one of the main directions of spinal surgery research, and with the emergence of new technologies and instruments, lumbar interbody fusion will be further developed.